# Primary chondrosarcoma presenting as an intrathoracic mass: A report of three cases

**DOI:** 10.3892/ol.2014.2275

**Published:** 2014-06-23

**Authors:** XUE-YUAN WANG, SU HU, LING-CHUAN GUO, JIE CHEN, MO ZHU, FEI-RONG YAO, CHUN-HONG HU

**Affiliations:** 1 Department of Radiology, The First Affiliated Hospital of Soochow University, Suzhou, Jiangsu 215006, P.R. China; 2 Department of Pathology, The First Affiliated Hospital of Soochow University, Suzhou, Jiangsu 215006, P.R. China; 3 Department of Radiology, The Affiliated Third Hospital of Suzhou University, Changzhou, Jiangsu 213003, P.R. China

**Keywords:** chondrosarcoma, imaging, computed tomography, roentgenogram, magnetic resonance imaging

## Abstract

Primary intrathoracic chondrosarcomas are rare tumors. The present study reports three cases of primary intrathoracic chondrosarcomas in two males and one female aged between 45 and 64 years. Clinically, one case presented with cough and blood sputum, while the other two cases of primary intrathoracic chondrosarcoma were found incidently during a routine health examination. Radiologically, the chondrosarcomas presented as large masses with intratumoral calcification. Chondrosarcoma should be distinguished from other calcified pulmonary lesions. In this study, all three cases underwent surgical treatment, and in one case, the surgery was accompanied by radiotherapy. To date, all patients have been followed up for between two and three years and are alive.

## Introduction

Accounting for approximately one-quarter of all primary osseous sarcomas, chondrosarcoma is the second most common type of primary sarcoma of bone. Although chondrosarcoma only occasionally originates within the lungs and bronchi, it is the most common type of primary malignancy of the chest wall (1). Chondrosarcomas usually present with an anterior location within the chest wall, arising from the costochondral arches or sternum. Those tumors that arise from the ribs occasionally manifest as intrathoracic masses with minimal osseous involvement. Chondrosarcomas in the lungs and bronchi are extremely rare (2). The majority of intrathoracic chondrosarcomas manifest clinically as painless or painful masses, and occasionally manifest as cough or chest pain. Surgery is considered the primary treatment of chondrosarcoma, occasionally accompanied by chemotherapy or radiation therapy (2). The present study describes the imaging findings of three cases of chondrosarcoma that presented as intrathoracic masses, with the aim to recognize the clinical and imaging features of this disease with regard to the current literature. Patients provided written informed consent.

## Case report

### Case 1

**Figure 1:**
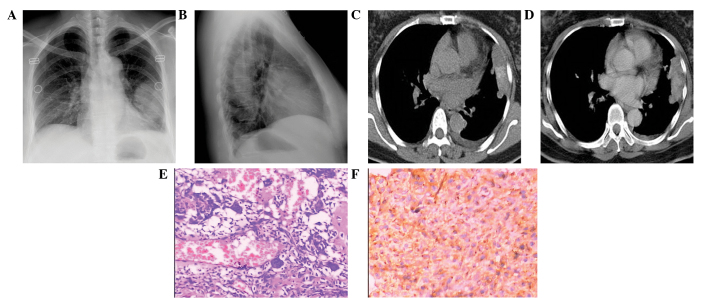
(A) Posteroanterior and (B) left lateral radiographs showing a round mass in the left upper lung lobe. (C) Non-contrast chest CT scan showing an oval mass with intratumoral calcification in the anterior segment of the left upper lobe. The mass was found to be closely attached to the adjacent pleura. (D) Chest CT scan with intravenous contrast showing heterogeneously enhanced mass. (E) Microscopic findings of the tumor showing pleomorphic tumor cells with areas of chondroid differentiation. Hematoxylin and eosin staining; magnification, ×100. (F) Immunohistochemistry showing positive cluster of differentiation (CD)99 expression in the tumor cells. CT, computed tomography.

A 52-year-old female presented to the First Affiliated Hospital of Soochow University (Suzhou, Jiangsu) with an intermittent cough and bloody sputum that had persisted for four months. A chest roentgenogram revealed a homogeneous, round, well-defined shadow in the left lung (Fig. 1A and B). Computed tomography (CT) revealed an irregular hyperdense lung mass with coarse intratumoral calcifications in the anterior segment of the left upper lobe. Part of the mass was closely attached to the pleura, but there was no cartilage or bone destruction (Fig. 1C). The mass was heterogeneously enhanced upon the administration of contrast medium (Fig. 1D). The mass was completely resected using a left thoracotomy and was easily separated from the parietal pleura. Grossly, the resected mass was white in color and measured 5×4×4 cm, with necrosis at the cut surface. Two small nodules could be observed on the surface of the pleura and diaphragm. The final pathological diagnosis was of a dedifferentiated chondrosarcoma (Fig. 1E). Immunohistochemically, the tumor was positive for S-100 in the cartilaginous component and cluster of differentiation (CD)99 in the sarcomatous component (Fig. 1F), but negative for smooth muscle actin, CD34, CD117, B-cell lymphoma-2 and cytokeratin 18. The patient received no further therapy. To date, the patient has been followed up for three years and is alive.

### Case 2

**Figure 2:**
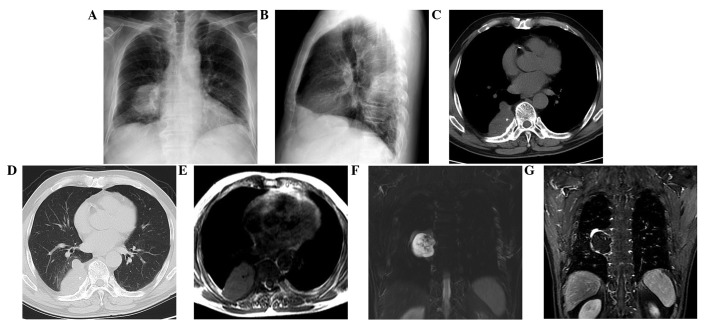
(A) Posteroanterior and (B) right lateral radiographs showing a round hyperdensity mass in the right lower lung lobe. (C and D) Non-contrast chest CT scan showing an oval mass with small calcifications in the posterior segment of the right lower lobe. The mass is located in the vicinity of the seventh rib and the T7 vertebral body. (E) T1-weighted axial MRI showing a right-lower-lobe mass with a signal intensity equal to that of the chest wall musculature. (F) T2-weighted coronal MRI showing that the signal intensity of the mass is similar to that of cerebrospinal fluid. (G) Contrast-enhanced T1-weighted coronal MRI showing that the mass is ring-like enhanced. CT, computed tomography; MRI, magnetic resonance imaging.

A 64-year-old male presented with a slightly hyperdense mass with rounded boundaries on the right lower lung, which had been incidentally diagnosed during a routine health examination. (Fig. 2A and B). A CT scan of the chest revealed that the mass was homogeneous and calcified, originating from the right chest wall and protruding into the thoracic cavity (Fig. 2C and D). Chest magnetic resonance imaging (MRI) revealed that the mass was slightly hypointense on the T1-weighted image (T1WI) (Fig. 2E) and hyperintense on the T2WI (Fig. 2F), with an enhanced periphery following the administration of intravenous contrast medium (Fig. 2G). The calcification was found to be hypointense on the T1- and T2WIs. A right thoracotomy revealed a mass on the inner surface of the right seventh rib that did not adhere to the lung parenchyma. The mass was completely excised and measured 3×4×4 cm. Grossly, the mass consisted of cyst fluid, gel-like tissue and a small quantity of cartilage tissue. Histopathological examination indicated that the mass was a grade 1 chondrosarcoma. Staining for S-100 and Ki-67 was positive, but staining for smooth muscle actin, desmin, vimentin and p53 was negative. The patient received standard radiotherapy (total of 50 Gy; 2 Gy per fraction) during the two years of follow-up and is alive at the time of writing.

### Case 3

**Figure 3:**
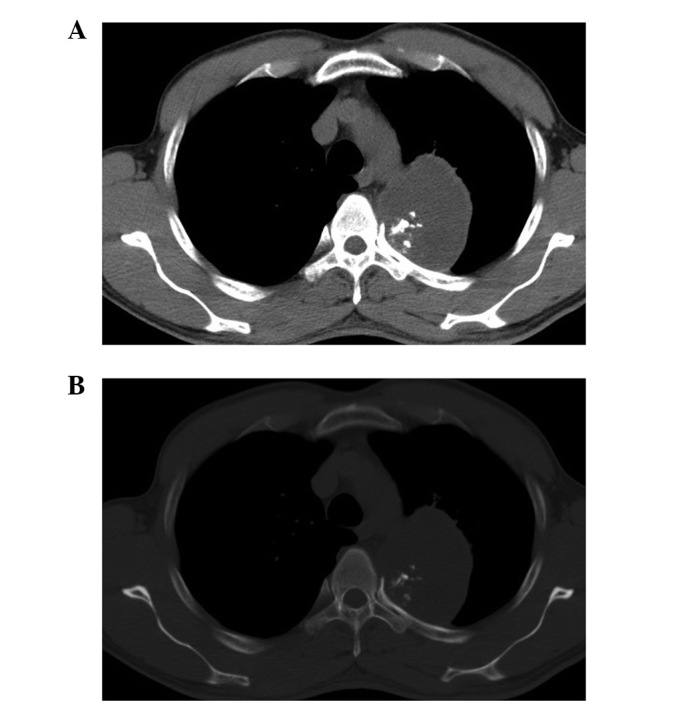
Non-contrast chest computed tomography (CT) scan showing an oval, near-water-attenuation mass with small scattered calcifications in the posterior segment of the right upper lobe. The mass is located near the fourth rib and the T4 vertebral body.

A 45-year-old male presented with a round mass on the left lung. The mass was homogeneously hypodense (near the density of water) and 6×5 cm in size on CT images (Fig. 3). The mass protruded into the thoracic cavity and there was a certain amount of bony destruction to the left fourth rib. Furthermore, the left upper lung was compressed. A left thoracotomy was performed and the mass was found to be adhered to the left fourth rib, with a wide base and areas of cartilage in the basilar section. Histopathological examination showed that the lesion was a grade 1 chondrosarcoma. The patient received no further treatment and is alive after three years of follow-up.

## Discussion

Primary chest chondrosarcomas, including those arising from the chest wall (bone, muscle and pleura) and lungs or bronchi, are uncommon (3). Primary pulmonary chondrosarcoma is extremely rare, with strict diagnositc criteria (4,5). The majority of chondrosarcomas arising from the chest wall appear as soft-tissue masses within the chest wall (2). Tumors presenting as intrathoracic masses are observed in certain cases of primary chest wall chondrosarcoma and all cases of primary pulmonary chondrosarcoma. The present study reported three cases of chondrosarcoma that presented as intrathoracic masses (1). None of the cases could be diagnosed as primary pulmonary chondrosarcoma based on the diagnostic criteria (4). However, they were considered to be primary intrathoracic chondrosarcoma due to the absence of a clinical history and other lesions.

The natural history of primary intrathoracic chondrosarcoma is similar to that of skeletal chondrosarcoma. Chondrosarcomas usually occur in individuals >30 years old, with a male predominance (1). Patients may be symptom-free during the initial phase when the chondrosarcoma grows slowly. The most common symptoms of intrathoracic chondrosarcoma include a non-productive cough, chest pain, dyspnoea and hemoptysis (5–8). The tumors are often locally invasive and have a high rate of recurrence (5,9).

The imaging features of primary intrathoracic chondrosarcoma resemble those of skeletal chondrosarcoma. CT imaging of primary intrathoracic chondrosarcoma often reveals a large soft-tissue mass with heterogeneous calcification and occasional destruction of the adjacent bone (5,6,8,10–12). Intratumoral calcification is one of the characteristic imaging findings of chondrosarcoma and may be an important indicator for diagnosis (13). The MRI features of chondrosarcoma show as isointense on the T1WI and hyperintense on the T2WI (12,13). However, in the present case, calcification was revealed as hypointense on the T1- and T2WIs. Atelectasis or obstructive inflammation may present when the bronchus is obstructed by the mass. Pleural effusion may be performed if the pleura is involved (14). Significant invasion to the pulmonary arteries has been reported (15). Atypical imaging features are found in certain cases and may mislead the diagnosis. Intratumoral necrosis or cystic degeneration has also been found in certain cases (6,14). Parker *et al* (12) reported a case of primary pulmonary chondrosarcoma with CT and MRI features mimicking a bronchogenic cyst.

Primary intrathoracic chondrosarcomas are usually indistinguishable using imaging methods. Primary intrathoracic chondrosarcomas should be differentiated from calcified pulmonary lesions, including pulmonary hamartoma (PH), primary lung cancer and other intrathoracic sarcomas (3,13). PH is the most benign lung tumor, with a relatively small size (diameter of ≤4 cm on chest radiograph or ≤2.5 cm on CT). The finding of fat and calcification together is diagnostic, as the tumor is composed of fat, epithelial tissue, fibrous tissue and cartilage. Furthermore, an air cleft on the side or the inside is characteristic of pulmonary hypertension. It is necessary to regularly follow-up hamartoma-like lesions, as chondrosarcoma may develop from persistent hamartomas (16). Primary lung cancer may present with calcification of various patterns as a result of a secretary function of the carcinoma, chemotherapy or hypercalcemia. Although calcification within lung cancer is rare, it is difficult to differentiate intrathoracic chondrosarcoma from primary lung cancer when intratumoral calcification is found. Primary intrathoracic chondrosarcomas are difficult to differentiate from other sarcomas, including Ewing’s sarcoma, primitive neuroectodermal tumors and osteosarcomas, if the diagnosis depends only on the imaging technique. An accurate diagnosis is possible by combining the imaging findings with the clinical presentation. For example, Ewing’s sarcoma and osteosarcoma often occur in young individuals <30 years old; however, chondrosarcomas occur in adults >30 years old.

In conclusion, primary intrathoracic chondrosarcoma is a rare, malignant tumor that originates from the chest and may involve the chest wall, lungs or bronchi. Radiologically, primary intrathoracic chondrosarcomas usually occur as large masses with intratumoral calcification. Although histological analysis is always required for a definite diagnosis, imaging is important for analyzing the tumor. It may also be useful to pay careful attention to the imaging features of the tumor and its clinical manifestations for the diagnosis.
